# Migration and allergic diseases in a rural area of a developing country

**DOI:** 10.1016/j.jaci.2016.01.052

**Published:** 2016-09

**Authors:** Alejandro Rodriguez, Maritza G. Vaca, Martha E. Chico, Laura C. Rodrigues, Mauricio L. Barreto, Philip J. Cooper

**Affiliations:** a Laboratorio de Investigación FEPIS, Quinindé, Esmeraldas Province, Ecuador; b Faculty of Epidemiology and Population Health, London School of Hygiene and Tropical Medicine, London, United Kingdom; c Facultad de Ciencias Medicas, de la Salud y la Vida, Universidad Internacional del Ecuador, Quito, Ecuador; d Centro de Pesquisas Gonçalo Muniz, FIOCRUZ, Salvador, Brazil; e Instituto de Saude Coletiva, Universidade Federal da Bahia, Salvador, Brazil; f Institute of Infection and Immunity, St George's University of London, London, United Kingdom

To the Editor:

Studies in developing countries (DCs) have frequently reported a lower prevalence of allergic diseases (ADs) in rural areas compared with urban settings, and this has been attributed to the protective effects of environmental exposures such as rural lifestyle.^1^ Recent evidence from studies conducted in Africa and Asia showed that ADs are increasing in urban and even in rural settings, reducing the urban-rural prevalence gap.^2^, ^3^ It has been hypothesized that temporal increases in the prevalence of ADs might be associated with urbanization processes, especially with the change from rural to more modern urban lifestyles.^1^

Migration is an important component of the urbanization process and involves socioeconomic, environmental, and lifestyle changes in rural and urban populations. However, the effects of migration on ADs in urban and rural settings of DCs have not been explored.^4^ The impact of migration on ADs has been largely investigated by comparing populations that have migrated from DCs (presumed low risk for ADs) to developed countries (presumed high risk).^5^ These studies have shown that being born in a country of low risk provides protection against asthma, but this protection may decline with the length of residence in the new environment.^5^ Others studies have shown that age of migration and time since migration are associated with the risk of asthma and other ADs, often leading to a higher risk of atopy and allergy among migrants than among the local population.^6^

The Social Changes, Asthma and Allergy in Latin America (SCAALA) study has been investigating the effects of migration on the prevalence of ADs in schoolchildren living in rural and urban areas.^4^ We studied 4295 rural and 2510 urban children aged 5 to 16 years attending a convenience sample of schools in Esmeraldas province, Ecuador. Data on potential risk factors, migration (direction and distance of migration, age at migration, and time since migration), and wheeze, rhinitis, and eczema symptoms within the previous 12 months were collected using an investigator-administered questionnaire that included the core allergy questions of the International Study of Asthma and Allergies in Childhood (ISAAC phase II).^4^ Atopy was measured by skin prick testing to 7 aeroallergens.

Results from the rural area showed that children who migrated during the first year of life had a greater risk of wheeze and rhinitis than did nonmigrant children and that children with a history of international migration (children from rural areas of Colombia) had a higher prevalence of rhinitis than did nonmigrant children (Table I). The study also evaluated the effects of maternal migration on allergic outcomes in children using the variables maternal history of migration and children living with one or no parent. These analyses suggested that children whose mothers had a history of migration had a greater risk of eczema compared with children whose mother did not, and children who did not live with any parent had more wheeze than did children living with both parents (Table I). The magnitude of the latter association was greater for all allergic symptoms among children of migrant mothers (Table II). No associations were observed for atopy (at least 1 positive allergen skin test result).

The present study is unique in investigating migrants within a rural area of a DC, where migrants come from urban and rural settings. In this setting, age at migration and international migration were important factors associated with a higher risk of ADs in rural populations. A novel observation was the effect of migrant status of the mother on the prevalence of ADs: children of migrant mothers not living with either parent had a 2-fold greater risk of all 3 ADs compared with children living with both parents. These data raise a question: *Could it be that social effects of migration, such as absence of parents at home, are important determinants of the increase in ADs in rural populations of DCs*? To answer this question, we need to consider some demographic patterns in these regions. It is well known that people in rural villages move to urban areas, temporally or permanently, in search of work to improve their quality of life. A high proportion of these rural migrants are single women who provide economic support for their families. Most of these women leave their children in the community of origin to be cared for by relatives. Some of these immigrants are able to settle in the city while others return to their rural communities.^7^ In the SCAALA rural population, 31% of the children and 23% of the mothers had a history of migration and 15% of the children lived with no parent.

If the absence of parents at home (especially the mother) is an important determinant of the increase in ADs in DCs, then 2 migration trends that have occurred over recent decades might help us understand temporal trends in ADs. In the past, most economic migrants were young men, but now “feminization of migration” is a growing trend worldwide because of a greater demand for female labor.^8^ Second, “circular migration*”* is a common phenomenon in regions that are undergoing high levels of urbanization, and it refers to repeated migrations between rural and urban areas due to improvements in transport and modern forms of communication.^9^

Migration affects not only the individuals who migrate but also their family. Migration impacts on roles, support structures, and responsibilities of family members, resulting in changes in social and psychological factors. In the case of maternal migration, children who remain in their community may experience heightened levels of stress and depression because of separation from their primary carer. Psychological mechanisms have been proposed to explain how emotional factors, in the context of family, might affect the development of allergic diseases.^10^ For this reason, we propose that the absence of the parents at home, through temporary or permanent migration, may contribute to the increase in ADs in rural and urban populations of DCs.

Finally, further analyses in different populations living in rural and urban areas evaluating the effects of migration on ADs are required. A better understanding of the social, psychological, and environmental effects of migration on ADs in DCs is required.

## Figures and Tables

**Table I:** ORs and 95% CIs for associations between migration variables and allergic symptoms adjusted for sex, age, and socioeconomic status

Variable	Category	N	OR (95% CI)		
Wheeze	Rhinitis	Eczema
Direction of migration	NM	2964	1	1	1
	Rural to rural	555	1.13 (0.84-1.52)	1.02 (0.7-1.49)	1.23 (0.82-1.83)
	Urban to rural	776	0.97 (0.74-1.27)	1.18 (0.86-1.61)	1.16 (0.81-1.66)
Distance of migration	NM	2964	1	1	1
	National	1263	0.99 (0.79-1.25)	1.04 (0.79-1.38)	1.21 (0.90-1.64)
	International	68	1.71 (0.88-3.32)	2.39 (1.16-4.92)∗	0.64 (0.16-2.66)
Age at migration (y)	NM	2964	1	1	1
	<1	269	1.47 (1.02-2.12)∗	1.59 (1.03-2.46)∗	1.25 (0.73-2.14)
	1-5	560	0.96 (0.71-1.31)	1.18 (0.83-1.69)	1.17 (0.78-1.75)
	>5	502	0.88 (0.62-1.24)	0.76 (0.48-1.19)	1.16 (0.75-1.79)
Time since migration (y)	NM	2964	1	1	1
	<3 vs NM	383	0.98 (0.68-1.4)	0.94 (0.6-1.49)	0.96 (0.57-1.61)
	3-5 vs NM	197	0.56 (0.31-1.02)	0.9 (0.48-1.69)	1.53 (0.86-2.7)
	>5 vs NM	751	1.21 (0.94-1.58)	1.26 (0.92-1.73)	1.21 (0.85-1.73)
Maternal history of migration	No	3314	1	1	1
	Yes	981	1.22 (0.96-1.53)	1.24 (0.93-1.65)	1.88 (1.39-2.53)∗
Parents living in the child's house	Both	2490	1	1	1
	One	1146	1.07 (0.84-1.36)	1.16 (0.87-1.54)	1.21 (0.88-1.67)
	None	659	1.57 (1.2-2.05)∗	1.29 (0.92-1.81)	1.27 (0.86-1.86)

*NM*, No migrant; *OR*, odds ratio.

Outcomes were defined as recent wheeze—reported wheezing during the previous 12 months; recent eczema—having a reported itchy rash with a flexural distribution in the previous 12 months; and recent rhinitis—nasal stuffiness or sneezing without a cold accompanied by itchy eyes in the previous 12 months.

∗ *P* < .05.

**Table II:** ORs and 95% CIs for associations between allergic symptoms and parents living in the child's home (live with parents) stratified by maternal history of migration∗

Allergic symptom	Live with parents	Maternal history of migration					
No			Yes		
OR∗	95% CI	*P* value	OR∗	95% CI	*P* value
Wheeze	One vs both	1	0.76-1.34	.976	1.2	0.77-1.87	.429
	None vs both	1.44	1.06-1.95	.02	2.17	1.25-3.77	.006
Rhinitis	One vs both	1.03	0.73-1.46	.858	1.46	0.85-2.52	.171
	None vs both	1.1	0.74-1.64	.627	2.07	1.05-4.08	.036
Eczema	One vs both	0.96	0.63-1.46	.857	1.63	0.95-2.77	.074
	None vs both	1.03	0.64-1.65	.916	2.12	1.07-4.17	.031

*OR*, Odds ratio.

∗ ORs adjusted for sex, age, and socioeconomic status.

## References

[1] von Hertzen L., Haahtela T. (2006). Disconnection of man and the soil: reason for the asthma and atopy epidemic?. J Allergy Clin Immunol.

[2] Addo-Yobo E.O.D., Woodcock A., Allotey A., Baffoe-Bonnie B., Strachan D., Custovic A. (2007). Exercise-induced bronchospasm and atopy in Ghana: two surveys ten years apart. PLoS Med.

[3] Selcuk Z.T., Demir A.U., Tabakoglu E., Caglar T. (2010). Prevalence of asthma and allergic diseases in primary school children in Edirne, Turkey, two surveys 10 years apart. Pediatr Allergy Immunol.

[4] Cooper P.J., Chico M.E., Vaca M.G., Rodriguez A., Alcântara-Neves N.M., Genser B. (2006). Risk factors for asthma and allergy associated with urban migration: background and methodology of a cross-sectional study in Afro-Ecuadorian school children in Northeastern Ecuador (Esmeraldas-SCAALA Study). BMC Pulm Med.

[5] Cabieses B., Uphoff E., Pinart M., Antó J.M., Wright J. (2014). A systematic review on the development of asthma and allergic diseases in relation to international immigration: the leading role of the environment confirmed. PLoS One.

[6] Rottem M., Szyper-Kravitz M., Shoenfeld Y. (2005). Atopy and asthma in migrants. Int Arch Allergy Immunol.

[7] (2005). Mujeres migrantes de América Latina y el Caribe: derechos humanos, mitos y duras realidades.

[8] Chammartin G. (2002). The feminization of international migration. Int Migr Program Int Labour Organ.

[9] Beguy D., Bocquier P., Zulu E.M. (2010). Circular migration patterns and determinants in Nairobi slum settlements. Demogr Res.

[10] Kaugars A.S., Klinnert M.D., Bender B.G. (2004). Family influences on pediatric asthma. J Pediatr Psychol.

